# *Wnt2bb* Induces Cardiomyocyte Proliferation in Zebrafish Hearts via the *jnk1/c-jun/creb1* Pathway

**DOI:** 10.3389/fcell.2020.00323

**Published:** 2020-05-25

**Authors:** Xiangwen Peng, Shunyang Fan, Jing Tan, Zhi Zeng, Meiling Su, Yuan Zhang, Ming Yang, Luoxing Xia, Xuejiao Fan, Weibin Cai, Wai Ho Tang

**Affiliations:** ^1^Guangzhou Women and Children’s Medical Centre, Institute of Pediatrics, Guangzhou Medical University, Guangzhou, China; ^2^Heart Center, The Third Affiliated Hospital of Zhengzhou University, Zhengzhou, China; ^3^Guangdong Engineering & Technology Research Center for Disease-Model Animals, Laboratary Animal Center, Zhongshan School of Medicine, Sun Yat-sen University, Guangzhou, China

**Keywords:** zebrafish heart regeneration, *wnt2bb*, *c-jun/jnk1/creb1*, nkx25, *c-jun*, *creb1*, *nkx2.5*

## Abstract

Previous studies have demonstrated that inhibition of canonical Wnt signaling promotes zebrafish heart regeneration and that treatment of injured heart tissue with the Wnt activator 6-bromo-indirubin-3-oxime (BIO) can impede cardiomyocyte proliferation. However, the mechanism by which Wnt signaling regulates downstream gene expression following heart injury remains unknown. In this study, we have demonstrated that inhibition of injury-induced myocardial *wnt2bb* and *jnk1/creb1/c-jun* signaling impedes heart repair following apex resection. The expression of *jnk1*, *creb1*, and *c-jun* were inhibited in *wnt2bb* dominant negative (dn) mutant hearts and elevated in *wnt2bb*-overexpresssing hearts following ventricular amputation. The overexpression of *creb1* sufficiently rescued the *dn-wnt2bb*-induced phenotype of reduced *nkx2.5* expression and attenuated heart regeneration. In addition, *wnt2bb/jnk1/c-jun/creb1* signaling was increased in *Tg(hsp70l:dkk1)* transgenic fish, whereas it was inhibited in *Tg(hsp70l:wnt8)* transgenic fish, indicating that canonical Wnt and non-canonical Wnt antagonize each other to regulate heart regeneration. Overall, the results of our study demonstrate that the wnt2bb-mediated *jnk1/c-jun/creb1* non-canonical Wnt pathway regulates cardiomyocyte proliferation.

## Introduction

Myocardial Infarction (MI) is a leading cause of morbidity and mortality in industrialized countries (Guo et al., 2016). The massive lost cardiomyocytes (CMs) resulting from MI cannot be supplemented due to the low proliferation ability of adult human heart cells, and a lack of an adequate number of CMs causes chronic overload and subsequent dysfunction that ultimately leads to heart failure and death (Hajduk et al., 2013). In contrast, the injured hearts of zebrafish and neonatal mice can be fully repaired through pre-existing CM dedifferentiation and proliferation (Poss et al., 2002; Lepilina et al., 2006). There are two important phenotypes of CM dedifferentiation. The first is reactivation of embryonic cardiac genes, such as *gata4, hand2, nkx2.5*, and *tbx5* in the myocardium surrounding the sites of injury (Lepilina et al., 2006; Kikuchi et al., 2010). The second phenotype of CM dedifferentiation requires the disassembly of sarcomeres (Jopling et al., 2010). However, the mechanism by which embryonic cardiac genes are reactivated and sarcomere disassembly is regulated remains unknown. Thus, further investigation into the mechanisms mediating embryonic cardiac gene reactivation is needed to promote the development of regenerative therapies in patients suffering from heart failure.

The Wnt pathway is one of the important pathways that regulate cardiac development, cardiovascular diseases, cardiac hypertrophy, MI, and heart failure, and the signal is evolutionarily conserved from *C. elegans* to mammals. The Wnt pathway has a biphasic role in heart development, where cardiac precursor cells require active canonical Wnt signaling, while cardiomyocyte differentiation requires inhibition of canonical Wnt signaling (Naito et al., 2006; Ueno et al., 2007; Tian et al., 2010). To date, accumulated evidence shows that Wnt signaling plays an important role in the adaptive response of the heart to heart disease, and many study have shown that interventions in Wnt signaling on cardiac remodeling have effects (Askevold et al., 2014; Matthijs Blankesteijn and Hermans, 2015; Francis Stuart et al., 2016; Hermans et al., 2016; Huisamen et al., 2016). However, although the role of the Wnt pathway in mediating CM proliferation during heart regeneration remains unclear, an understanding of the associated mechanism promote the development of treatments for heart disease.

Recent studies have reported that the use of small molecule inhibitors of Wnt improves CM proliferation during zebrafish heart regeneration (Xie et al., 2019; Zhao et al., 2019). Wnt signaling is divided into two pathways, the β-catenin-dependent and -independent canonical and non-canonical Wnt pathways, respectively (Kwon et al., 2007; Macdonald et al., 2007; Semenov et al., 2007). Although canonical and non-canonical Wnt signaling have different functions, accumulating evidence has suggested a role for the dynamic balance between canonical and non-canonical Wnt signaling in cardiac formation and differentiation (Ai et al., 2007; Kwon et al., 2007; Han et al., 2016). Taken together, these findings suggest that inhibition of canonical Wnt signaling may increase non-canonical Wnt signaling during heart regeneration. In this study, we showed that the expression of the genes *jnk1, c-jun*, and *creb1*, which are involved in the non-canonical Wnt pathway (Sanyal et al., 2002; Yamanaka et al., 2002; Oishi et al., 2003; Sabapathy et al., 2004; Chen et al., 2005; Semenov et al., 2007; Farías et al., 2009; Lee et al., 2010), were induced during zebrafish heart regeneration and were required for cardiomyocyte proliferation. The embryonic cardiac gene *nkx2.5* is under the control of *jnk1/c-jun*/*creb1* signaling, and this signaling is induced by the *wnt2bb* protein. The results of our study indicate that the *wnt2bb*/*jnk1/c-jun*/*creb1* pathway is a potential therapeutic target for the treatment of heart disease.

## Methods

### Zebrafish Strains and Ventricular Resection

Adult zebrafish (4–12 months of age) were used for ventricular resection surgery as previously described (Poss et al., 2002). Briefly, 20% of the ventricular muscle was removed at the apex with iridectomy scissors. The transgenic zebrafish lines used in this study included *Tg(hsp70l:wnt8)* (Ueno et al., 2007) and *Tg(hsp70l:dkk1b)* (Ueno et al., 2007). To generate the transgenic fish *Tg(hsp70l:dn-creb1-E2A-mCherry;Cryoaa:GFP), Tg(hsp70l:dn-c-jun-E2A-mCherry;Cryoaa:GFP), Tg(hsp70l:wnt2 bb-ORF-E2A-mCherry;Cryoaa:GFP)*, *Tg(hsp70l:dn-wnt2bb-E2A- GFP;Cryoaa:mCherry), Tg(hsp70l:creb1ORF-E2A-mcherry;Cryo aa:GFP), Tg(hsp70l:c-junORF-E2A-mcherry;Cryoaa:GFP)*, and *Tg(hsp70l:dn-wnt2-E2A-mcherry;Cryoaa:GFP)*, the transgene was engineered using Gateway technology (Life Sciences). The plasmid pDestTol2pA2 was used as a destination vector, and 100 ng/μL of this plasmid) was injected into one-cell stage wild-type zebrafish embryos together with 100 ng/μL of Tol2 transposase mRNA (Kwon et al., 2007). For each fish line, we obtained 3–6 founders, and one founder was verified for the intended effects by confirming phenotypes and was subsequently propagated.

### Genotyping Assay

Two methods were used for transgenic fish genotyping assays. In the first method, genotyping the allele was performed based on fluorescence microscopy by heat shocking fish at 37°C for 2 h and then detecting the GFP or RFP expression in the whole body and eyes at 60 hpf (Supplementary Figure S3). In the second method, genomic DNA from the transgenic fish was isolated using a Tissue DNA Kit (Omega). Subsequently, the construct was detected by PCR using the forward primer 5′-GACAGGACTTTTTCCCCGAC-3′ and gene-specific reverse primer. The forward primer was located at the end of the *hsp70l* promoter, while the reverse primer was located in the genes c-jun, jnk, and *wnt2bb*. For the *dn-c-jun, dn-creb, dn-wnt2bb*, and *dn-wnt2* fish, we used the mCherry reverse primer (5′-aactccttgatgatggccatgttg-3′) or the GFP reverse primer (5′-aacttgtggccgtttacgtcg-3′). Subsequently, the amplified fragments were sequenced to confirm the correctness of the constructs. The fish overexpressing *dkk* and *wnt8* were identified by their embryonic phonotype. We crossed the *dkk* and *wnt8* fish with the AB strain and heat shocked the *dkk* or *wnt8* embryo at the tailbud stage at 37°C for 2 h. After 2 days, the *hsp70l*:*dkk* embryos exhibited small and short tails and the *hsp70l:wnt8* embryos had no eyes.

For the heat-shock experiment, the temperature of the water harboring zebrafish was gradually increased to 37°C and then held for 1 h. After the heat shock, the transgenic fish were confirmed by observing the expected fluorescence in the eyes, whole body or heart, including the *wnt2bb, dn-creb, dn-c-jun*, and *dn-wnt2bb* fish (Supplementary Figures S4A–D’). We also performed qPCR to quantitatively assess the expression efficiency, including for the *c-jun, dn-wnt2bb*, and *dn-creb1* fish (Supplementary Figures S4E,F).

### Euthanasia

The fish were euthanized with an overdose of tricaine methane sulfonate (MS222, 200–300 mg/l) by prolonged immersion. Fish were left in the solution for at least 10 min following the cessation of opercular movement.

### Statistical Analysis

All experiments were performed with at least two biological replicates, and each group consisted of 4–7 samples. All values are presented as the means ± SEM, with the number of independent experiments stated in the respective figure legends. Significance was analyzed using two tailed Student’s *t*-test between groups, and differences were determined to be significant when ^∗^*P* < 0.05, ^∗∗^*P* < 0.01, ^∗∗∗^*P* < 0.001, ^****^P < 0.0001 (ns, not significant).

### Quantitative Reverse Transcription PCR and *in situ* Hybridization Analyses

Total mRNA was extracted from uninjured, 4 days post amputation (dpa) and 7 dpa and used for cDNA synthesis with a PrimeScript^TM^ II reagent kit (Takara, RR037A). The cDNA was then used for quantitative reverse transcription PCR (RT-qPCR) with a TB Green Premix Ex Taq^TM^ II kit (RR820A Takara, Japan) and primers specifically designed for *wnt2bb, c-jun, creb1, jnk1*, and *nkx2.5*. β-Actin expression was used for normalization. A dn-creb1 allele containing a mutation in Ser13 (serine-133 to alanine) (Dworkin et al., 2007), the dominant negative mutant of *c-Jun (dn-c-Jun)*, TAM67, was generated by removing the transactivation domain (amino acids 3-122) of the wild-type c-Jun gene by PCR. The *dn-c-Jun* allele encodes the DNA-binding domain of the wild-type *c-Jun* (Suto et al., 2004). As previously described (Hoppler et al., 1996), we generated a new wnt2bb-truncation dominant negative mutation [*dn-wnt2bb(*Δ*278-396)*] with a deleted portion of the *wnt1* domain. *In situ* hybridization analyses were performed on cryosections of hearts as previously described (Lepilina et al., 2006). PCR fragments of *c-jun, wnt2bb, creb1*, and *jnk1* were subcloned into the vector pGEM-T (Promega, Madison, WI, United States). Digoxigenin-labeled cRNA probes were transcribed using T7 and SP6 RNA polymerases (Invitrogen AM1322, Carlsbad, CA, United States). *In situ* signals were detected using anti-digoxigenin-AP (Roche 11093274910), visualized with NBT/BCIP substrates (Roche 11681451001) and imaged on a Nikon Eclipse Ni microscope equipped with a Nikon Digital Sight DS-Ri1 camera (Nikon, Tokyo, Japan). For each biological sample, 3–5 biological replicates and 3 technical replicates were used for RT-qPCR.

### Histology and Immunofluorescence Methods

Injured hearts were extracted, fixed and cryosectioned. Fibrin and scar analyses were performed by acid fuchsin orange G (AFOG) staining as previously described (Poss et al., 2002). Briefly, sections were hydrated and incubated in Bouin’s solution (Sigma HT10132) containing 5% acetic acid (Sigma A6283), 9% formaldehyde (Sigma F8775), and 0.9% picric acid (Sigma 197378). Slides were then placed in an AFOG solution containing aniline blue (Fisher 28631-66-5), orange G (Sigma O7252-25G), and acid fuchsin (Sigma F8129-25G). Myofibril, fibrin, and collagen were visualized as orange, red and blue, respectively. CM proliferation experiments were performed as previously described (Fang et al., 2013). An appropriate fish density (one fish/500 ml) was used to examine heart regeneration. The primary antibodies used in this study included anti-Mef2C (Santa Cruz sc-313; 1:100), anti-creb1 (Abcam ab31387; 1:200), anti-nkx2.5 (Abcam ab32096; 1:200), anti-jnk1 (Abcam ab179461; 1:200), anti-proliferating cell nuclear antigen (PCNA) (Sigma P8825; 1:200), anti-c-jun (Abcam ab31419; 1:200), and anti-MHC (DHSB MF20; 1:200). Secondary antibodies included goat anti-mouse Alexa-Fluor-488 (Invitrogen A-11001), goat anti-mouse Alexa-Fluor-555 (Invitrogen A-11001), goat anti-rabbit Alexa-Fluor-488 (Invitrogen A-11008) and goat anti-rabbit Alexa-Fluor-555 (Invitrogen A-27039). For each heart, Z-stacks were acquired from 10 μm-thick sections using a Zeiss Axion Observer. To quantify CM proliferation, three sections containing the injury border zone from each heart were used for apotome imaging with a 10 × objective. The number of Mef2c+ and PCNA+ Mef2c+ cells in the injury border zone were enumerated using ImageJ2. The percentages of PCNA+ Mef2c+ /Mef2c+ cells were calculated and summed to determine the CM proliferation index values. For each biological sample, 4–7 hearts were used for immunostaining and CM proliferation experiments. The MAGnify^TM^ Chromatin Immunoprecipitation System was used for the CHIP experiment. A 329-bp PCR product was generated using the primers cre2F (GTGCTGTGAAATAGCTCCTG) and cre2R (GCAGAATGAAGCCAGTGC). Confocal images were obtained using a Zeiss LSM 710 microscope.

To quantify gene expression, three sections containing the injury border zone from each heart were used for apotome imaging with a 10 × objective. The fluorescence Integrated Density for *wnt2bb*+, *c-jun*+, *jnk1*+, *creb1*+, and *nkx2.5*+ CMs at the injury border zone was determined using ImageJ2 software. For each biological sample, 4–7 hearts were used for immunostaining experiments.

## Results

### Non-canonical Wnt Signaling Is Upregulated During Zebrafish Heart Regeneration

**FIGURE 1:**
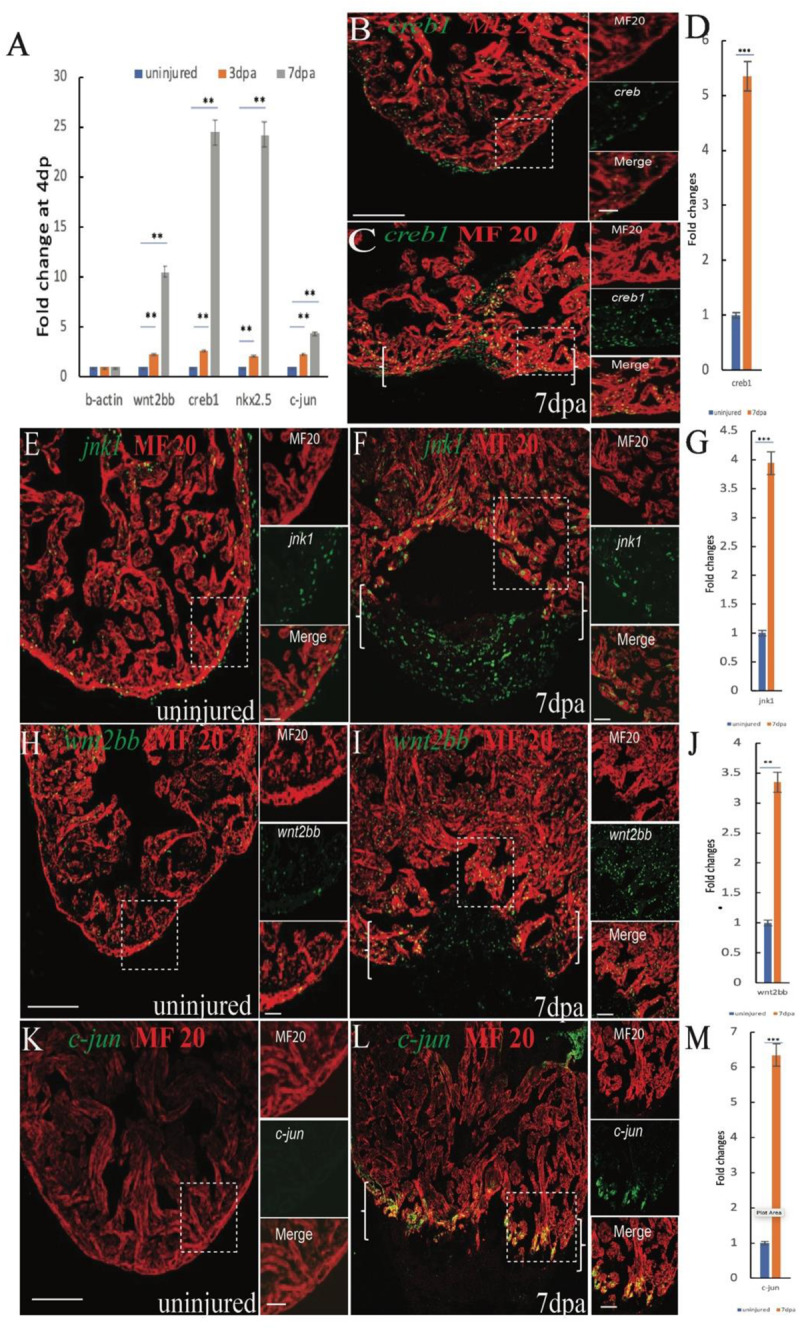
Induction of *jnk1, creb1*, *wnt2bb* and *c-jun* expression in injured cardiomyocytes during zebrafish heart regeneration **(A)** RT-qPCR results showing changes in *wnt2bb creb1 nkx2.5* and *c-jun* expression at 4 and 7 dpa. **(B,C)** Confocal microscopy images showing *creb1* expression during heart regeneration. At 7 dpa, *creb1* expression expanded to cardiomyocytes adjacent to the injury site. Green, *creb1*; Red, MHC. **(E,F)** Confocal microscopy images of *jnk* expression after cardiac injury. The expression of *jnk* was low in uninjured ventricles, but at 7 dpa, *jnk* expression was enhanced and expanded into the new cardiomyocytes at the wound site (arrows). Green, *jnk*; Red, MHC. **(H)** Whereas *wnt2bb* expression was detectable on the uninjured ventricle **(I)**, *wnt2bb* expression was enhanced in CMs adjacent to the injury site, and some non-CMs expressed *wnt2bb* at the apical edge of the wound at 7 dpa. Green, *wnt2bb*; Red, MHC. **(K,L)** Immunostaining analyses showed increased *c-jun* expression at the apical cell edges of the wounded heart and gradually increased at 7 dpa. Green, *c-jun*; Red, MHC. **(D,G,J,M)**. Bar graph showing the fold changes in *creb1*, *c-jun*, *jnk1*, and *wnt2bb* expression in cardiomyocyte at 7 dpa compared to that observed in the uninjured heart. The data are presented as the means ± SEM, and significance was determined using Student’s *t*-test. *n* = 5. Brackets indicate the amputation area. Boxes correspond to the magnified region in the adjacent panels [Scale bars: 100 μm (left) and 25 μm (right)].

Previous data showed that inhibition of canonical Wnt signaling enhances heart regeneration, and accumulating evidence has suggested a role for the dynamic balance between canonical and non-canonical Wnt signaling in cardiac formation and differentiation, indicating that non-canonical Wnt signaling may be essential for heart regeneration. To identify pathways associated with heart regeneration we performed RT-qPCR analyses to examine gene expression in the non-canonical Wnt pathway (Semenov et al., 2007) after cardiac amputation surgery. Interestingly, the expression of *jnk1, c-jun, creb1*, and *wnt2bb* mRNA in adult zebrafish hearts was gradually increased at 4 and 7 dpa (Figure 1A). To confirm the RT-qPCR data, we performed *in situ* hybridization (ISH) analyses for *creb1, c-jun, wnt2bb, and jnk1* and observed that the expression of *creb1, jnk1* and *c-jun* mRNA increased in cardiomyocytes near the injury site at 7 dpa (Supplementary Figures S1A–F). The immunostaining results showed that the expression of *jnk1, c-jun*, and *creb1* protein was also significantly increased in the CMs near the injury site (Figures 1B–G;K–M). c-Jun N-terminal kinases (JNKs) were originally identified as kinases that bind and phosphorylate c-jun on Ser-63 within its transcriptional activation domain. The cAMP response element-binding (CREB)1 proteins are activated upon phosphorylation by various kinases, including PKA, which phosphorylates CREB1 at serine 133, and *c-jun* functions upstream of CREB1 (Sanyal et al., 2002). We performed immunostaining with a PKA antibody and showed that PKA was activated in non-cardiomyocytes post apex amputation (Supplementary Figures S5A–C). Therefore, we speculated that the *creb1* activity in CMs may be activated by *jnk1/c-jun*, not PKA. These observations suggest that the non-canonical Wnt *jnk1/c-jun/creb1* pathway functions in cardiac regeneration.

The *jnk1, c-jun*, and *creb1* proteins are involved in multiple signaling pathways and are also regulated by non-canonical Wnt signaling. The results of previous studies indicated that inhibiting the canonical Wnt promotes zebrafish heart regeneration (Xie et al., 2019; Zhao et al., 2019), suggesting that the elevated *jnk1/c-jun/creb1* signaling may be induced by the non-canonical Wnt pathway. Previous data showed that *Wnt2* regulates myocardial cell differentiation through the non-classical Wnt pathway (Onizuka et al., 2012), suggesting that *wnt2 or wnt2bb* may mediate CM differentiation during zebrafish heart regeneration. The RT-qPCR results showed that *Wnt2bb* expression was induced at 4 and 7 dpa (Figure 1A), and the ISH results indicated that *wnt2bb* mRNA levels were increased in the CMs near the injured site during heart repair (Supplementary Figures S2A–D). Immunofluorescence results also indicated that the wnt2bb protein is induced in the CMs of the injury border zone during heart regeneration at 7 dpa (Figures 1H–J). These results indicated that wnt2bb mRNA and protein levels are elevated during heart regeneration.

### *wnt2bb* Is Required for Zebrafish Heart Regeneration

**FIGURE 2:**
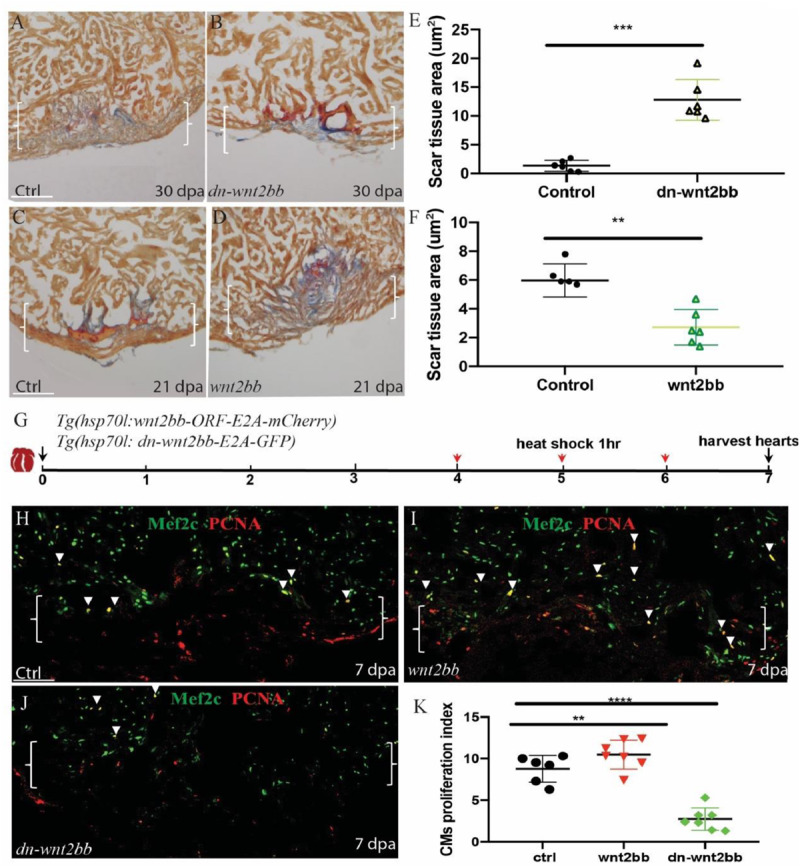
*Wnt2bb* mediates zebrafish heart regeneration. **(A,B)** Acid fuchsin orange G (AFOG) staining analyses reveal defective wound healing after ventricular resection in *Tg(hsp70l:dn-wnt2bb)* fish compared to that observed in the wild-type (ctrl) fish. Red arrow, fibrin; Blue, collagen; HS, heat shock of *Tg(hsp70l:dn-wnt2bb)* fish at 37°C for 1 h every 2 days for 30 days. **(E)** Bar charts showing the quantification of AFOG staining from **(A,B)**. **(C,D,F)** AFOG staining and quantification reveals increased wound healing after ventricular resection in *Tg(hsp70l:wnt2bb)* fish compared to that observed in the wild-type (ctrl) fish. Red arrow, fibrin; Blue, collagen; HS, heat shock of *Tg(hsp70l:wnt2bb)* fish at 37°C for 1 h every 2 days for 21 days. Six hearts were assayed per group. **(H–J)** Confocal microscopy image analyses of PCNA^+^Mef2C^+^ cells (arrowheads) in heat-shocked *Tg(hsp70l:dn-wnt2bb), Tg(hsp70l:wnt2bb)* and wild-type (ctrl) fish at 7 dpa. Green, PCNA; Red, Mef2C. Boxes correspond to the magnified region. **(K)** Bar charts showing the quantification of PCNA-labeled CM proliferation index values for heat-shocked, injured wild-type (ctrl) and *Tg(hsp70l:dn-wnt2bb) and Tg(hsp70l:wnt2bb)* fish hearts. **(G)** Schematic of the experimental plan to analyze PCNA/Mef2c proliferation after apex amputation. The data are presented as the means ± SEM from 5 to 7 hearts for each group. The proliferation data were collected for 4–6 sections per heart and averaged to generate each data point. Error bars: ± 1 SD. Significance was determined using Student’s *t*-test: **P < 0.01, ****P < 0.0001. Brackets indicate the amputation area. Ctrl, control. Scale bar, 100μm.

To study the role of *wnt2bb* in zebrafish heart regeneration, we generated wnt2bb-overexpressing transgenic fish *Tg(hsp70l:wnt2bb-ORF-E2A-mCherry)* and *Tg(hsp70l:dn-wnt2bb-E2A-GFP) wnt2bb* dominant negative mutant transgenic zebrafish. The wnt2bb fish have GFP-expressing eyes and express RFP following wnt2bb protein post heat shock (Supplementary Figures S4C,C’), and we also detected GFP expression after heat shock in dn-wnt2bb hearts (Supplementary Figures S4B,B’). Ventricular resection surgery was performed on the transgenic and wild-type fish, and the injured fish were heat-shocked 21 and 30 days, respectively, and the hearts were assayed for fibrin and collagen scars by AFOG staining. We observed that the heat-shocked control hearts largely contained cardiac muscle with minimal fibrin in the injury sites at 30 dpa, whereas the dn-*wnt2bb*-overexpressing heat-shocked fish had ventricular wounds that retained large patches of fibrin and some collagen (Figures 2A,B,E). In contrast, the *wnt2bb*-overexpressing heat-shocked fish showed minimal fibrin 21 dpa, but the control hearts contained numerous collagen scars (Figures 2C,D,F). Furthermore, we also we generated *Tg(hsp70l:dn-wnt2-E2A-GFP) wnt2* dominant negative mutant transgenic zebrafish. The AFOG staining results showed that heart regeneration in the *dn-wnt2* fish was normal. Although *dn-wnt2* and *dn-wnt2bb* retain only the wnt1 binding domain, their functions appear to be different. These results suggest that wnt2bb rather than wnt2 regulates cardiac regeneration (Supplementary Figures S5D–F).

In the PCNA/Mef2c proliferation assay, the *wnt2bb, dn-wnt2bb* and control fish were heat-shocked 1 h per day from 4 to 6 dpa, and the hearts were harvested at 7 dpa (Figure 2G). Consistent with the AFOG results, the PCNA/Mef2c proliferation index values of the *dn-wnt2bb* hearts decreased 63% compared the control fish (Figures 2H,J,K). The CM proliferation index value increased 25% in the CMs of the *Wnt2bb*-overexpressing fish (Figures 2H,I,K). These data indicate that *wnt2bb* is necessary for zebrafish heart regeneration.

### *c-jun/creb1* Signaling Regulates Injury-Induced Cardiomyocyte Proliferation

**FIGURE 3:**
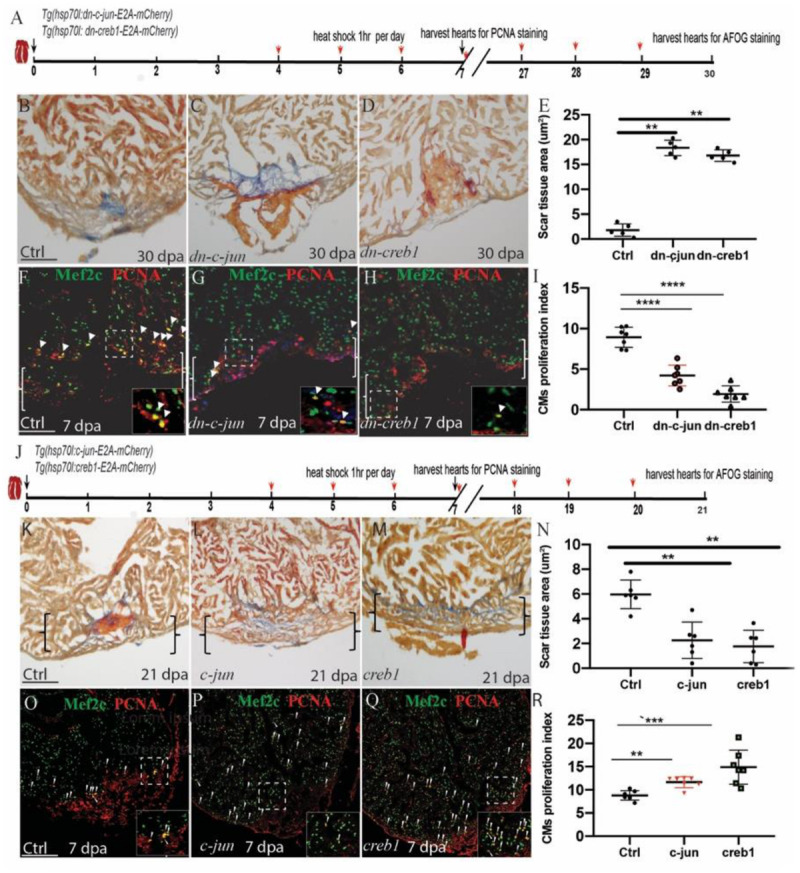
Regulation of injury-induced cardiomyocyte proliferation via *c-jun/creb1* signaling. **(A,J)** Schematic of the experimental plan to analyze PCNA/Mef2c proliferation and AFOG staining after apex amputation. **(B–E)** AFOG staining assays and quantification showing the apical wound after ventricular resection in wild-type (ctrl), *Tg(hsp70l:dn-c-jun)*, *and Tg(hsp70l:dn-creb1)* fish at 30 dpa. Red, fibrin; Blue, collagen. HS: heat shock at 37°C for 1 h. **(F–H)** Confocal microscopy image analyses of PCNA^+^Mef2C^+^ cells (arrowheads) in heat-shocked *Tg(hsp70l:dn-c-jun), Tg(hsp70l:dn-creb1)*, and wild-type (ctrl) fish at 7 dpa. Green, PCNA; Red, Mef2C. **(I)** Bar charts showing the quantification of PCNA-labeled CM proliferation index values for heat-shocked, injured wild-type (ctrl), *Tg(hsp70l:dn-c-jun)*, and *Tg(hsp70l:dn-creb1)* fish hearts. **(K–N)** AFOG staining analyses and quantification revealing defective wound healing after ventricular resection in *Tg(hsp70l:creb1) and Tg(hsp70l:c-jun)* fish compared to that observed in wild-type (ctrl) fish. Red arrow, fibrin; Blue, collagen; HS, heat shock at 37°C. **(O–Q)** Confocal microscopy image analyses of PCNA^+^Mef2C^+^ cells (arrowheads) in *Tg(hsp70l:creb), Tg(hsp70l:c-jun)*, and wild-type (ctrl) fish at 7 dpa. **(R)** Bar charts showing the quantification of PCNA-labeled CM proliferation index values for control, *Tg(hsp70l:creb1), Tg(hsp70l:c-jun)* fish at 7 dpa. The data are presented as the means ± SEM from 5 to 7 hearts for each group. For CM proliferation index experiments, proliferation data were collected for 4–6 sections per heart and averaged to generate each data point. Error bars: ± 1 SD. Significance was determined using Student’s *t*-test: **P < 0.01, ***P < 0.001, ****P < 0.0001. Scale bar, 100 μm. Ctrl, control. Boxes correspond to the magnified region.

To examine the role of *creb1/c-jun* in heart regeneration, we created *creb1* and *c-jun* dominant negative mutant transgenic zebrafish [*Tg(hsp70l:dn-creb1-E2A-mCherry)* and *Tg(hsp70l:dn-c-jun-E2A-mCherry)*, respectively]. These fish express GFP in their eyes and express RFP after heat shock together with dn-creb1 or dn-c-jun protein (Supplementary Figures S3A,B, S4A,A’,D,D’). We also observed that dn-creb1 overexpression in early embryo caused tailless defects as previously described. We performed ventricular resection surgery on the transgenic and wild-type fish, and the injured fish were heat-shocked 1 h per day from 4 to 29 dpa, and the hearts were harvested at 30 dpa (Figure 3A) and assayed for fibrin and collagen scars by AFOG staining. We observed that the heat-shocked heart injury sites of the control fish largely contained cardiac muscle with minimal fibrin at 30 dpa, while the heat-shocked *dn-c-jun*-overexpressing fish had ventricular wounds that retained large patches of fibrin and some collagen (Figures 3B,C,E). Similar results were observed for the *dn-creb1* transgenic zebrafish (Figures 3B,D,E). To understand the role of *creb1* and *c-jun* in CM proliferation, we overexpressed *dn-creb1* and *dn-c-jun* mutants from 4 to 6 dpa by heat shock for 1 h per day at 37°C. The cardiomyocyte proliferation index values were determined by immunostaining the cells that were positive for both and Mef2c (a cardiomyocyte nuclear marker). We observed that the cardiomyocyte proliferation index values for the *dn-c-jun* and *dn*-*creb1* mutant zebrafish decreased by 76 and 67%, respectively, compared to that observed in the wild-type hearts at 7 dpa (Figures 3F–I).

To examine the role of the *c-jun/creb1* pathway during zebrafish heart repair, we generated *Tg(hsp70l:creb1ORF-E2A-cherry)* and *Tg(hsp70l:c-junORF-E2A-cherry)* transgenic fish. We heat-shocked the fish for 1 h per day from 4 to 6 dpa, harvested the hearts and performed the PCNA/Mef2c proliferation assay at 7 dpa. The proliferation index values of the two transgenic fish lines increased by 16 and 20% over that observed in the hearts of the wild-type fish at 7 dpa (Figures 3O–R). We also performed ventricular resection surgery on the transgenic and wild-type fish, after which the injured fish were heat-shocked from 4 to 20 dpa, and the hearts were harvested at 21 dpa and then assayed for fibrin and collagen scars by AFOG staining. We observed that in the *c-jun*- *and creb1*-overexpressing the injured heart sites largely contained cardiac muscle with minimal fibrin at 21 dpa (Figures 3J,M,N), whereas the ventricular wounds of the heat-shocked wild-type fish retained large patches of fibrin and some collagen (Figures 3K,M,N). Overall, these data indicate that *c-jun/creb1* signaling is required for zebrafish heart regeneration.

As we used a heat-shock promoter, protein overexpression throughout the whole body could affect non-cardiac tissue. Therefore, we performed the ISH assays and observed that the expression of the endocardium marker *raldh2* (Supplementary Figures S6A,B) and the fibroblast marker *fn1* (Supplementary Figures S6C,D) did not significantly change in dn-wnt2bb fish. We also generated *Tg(hsp70l:dn-wnt2bb;tcf21:GFP)* double transgenic fish, and observed that the epicardium in these fish was not significantly affected (Supplementary Figures S6E,F). In addition, we performed qPCR with samples from dn-creb1, dn-wnt2bb, and c-jun-overexpressing fish and observed that the expression of the cardiac markers gata4 and nkx2.5 was inhibited in the dn-wnt2bb and dn-creb1 fish but increased in the c-jun-overexpressing fish (Supplementary Figure S4E). These data indicate wnt2bb signaling primarily occurs in cardiac myocytes.

### *jnk1/c-jun/creb1* Signaling Is Induced by the *Wnt2bb* Protein

**FIGURE 4:**
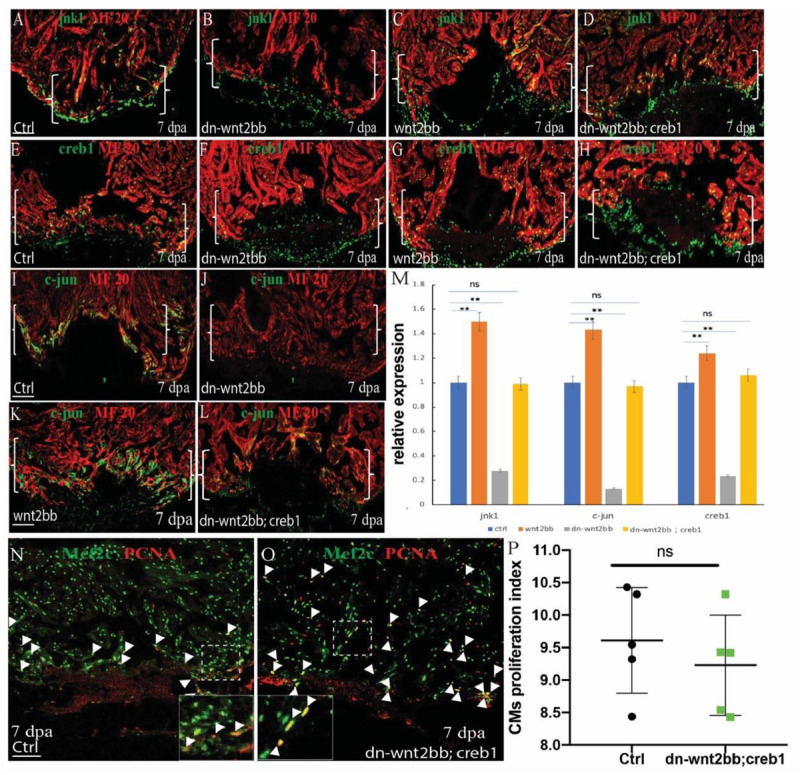
*wnt2bb* functions upstream of the *c-jun/creb* pathway. **(A–D)** Immunostaining analyses showing *jnk1* expression at wounded myocardial cell edges in the control hearts. **(A)** *Jnk1* expression decreased in heat-shocked *Tg(hsp70l:dn-wnt2bb)* fish hearts at 7 dpa **(B)** and increased in heat-shocked *Tg(hsp70l:wnt2bb)* fish hearts. **(C)** *jnk1* expression rescue of *Tg(hsp70l:dn-wnt2bb;hsp70l:creb1)* double transgenic fish and **(D)** control fish heat-shocked at 37°C for 1 hr daily from 4 to 6 dpa. **(E–H)** Confocal image analyses showing CREB1 expression at the wounded myocardial cell edges in control fish hearts **(E)**, elevated CREB expression in injured *Tg(hsp70l:wnt2bb)* fish hearts **(G)**, decreased CREB expression in *Tg(hsp70l:dn-wnt2bb)* fish hearts **(F)**, and in rescued *Tg(hsp70l:dn-wnt2bb;hsp70l:creb1)* double transgenic fish **(H)**. **(I–L)** Confocal microscopy image analyses showing *c-jun* expression at wounded myocardial cell edges in control fish hearts **(I)**, elevated c-jun expression in injured *Tg(hsp70l:wnt2bb)* fish hearts **(K)**, decreased *c-jun* expression in *Tg(hsp70l:dn-wnt2bb)* fish hearts **(J)**, and in rescued *Tg(hsp70l:dn-wnt2bb;hsp70l:creb1)* double transgenic fish **(L)**. **(N,O)** Confocal microscopy image analyses of PCNA^+^Mef2C^+^ cells (arrowheads) in heat-shocked *Tg(hsp70l:dn-wnt2bb;hsp70l:creb1)* and wild-type (ctrl) fish at 7 dpa. Green, PCNA; Red, Mef2C. **(M)** Bar charts showing the quantification of PCNA-labeled CM proliferation index values for heat-shocked, injured wild-type (ctrl), *Tg(hsp70l:dn-wnt2bb), Tg(hsp70l:wnt2bb)*, and *Tg(hsp70l:dn-wnt2bb;hsp70l:creb1)* fish hearts. Proliferation data were collected for 4–6 sections per heart and averaged to generate each data point. Green, PCNA; Red, Mef2C. **(P)** Bar charts showing the quantification of relative *nkx2.5* expression in heat-shocked, injured wild-type (ctrl), *Tg(hsp70l:dn-wnt2bb), Tg(hsp70l:wnt2bb)*, and *Tg(hsp70l:dn-wnt2bb;hsp70l:creb)* fish hearts. The data from five hearts in each group are presented. Error bars: ± 1 SD. Significance was determined using a Student’s *t*-test: **P < 0.01. *Tg(hsp70l:wnt2bb), Tg(hsp70l:dn-wnt2bb), Tg(hsp70l:dn-wnt2bb;hsp70l:creb1)* and control fish were heat-shocked at 37°C for 1 h daily from 4 to 6 dpa. Brackets indicate the amputation planes. Dotted line brackets indicate the amputation area. Scale bar, 100 μm. Green: *jnk1* **(A–D)**, *c-jun* **(E–H)**, and *creb1* **(I–L)**; Red, MF20. Fluorescence intensities were measured at the injury border zone using ImageJ. Boxes correspond to the magnified region.

c-Jun N-terminal kinases were originally identified as kinases that bind and phosphorylate *c-jun* on Ser-63 within its transcriptional activation domain. CREB proteins are activated by phosphorylation of the Ser-133 residue by *c-jun*, which functions upstream of *creb1*. To examine whether *jnk1/c-jun/creb1* signaling is mediated by *wnt2bb* during zebrafish heart regeneration, the expression of *jnk1, c-jun* and *creb1* was observed by immunofluorescence in the control, *hsp70l:dn-wnt2bb*, and *hsp70l:wnt2bb* transgenic fish. A rescue experiment was also performed in the *Tg(hsp70l:dn-wnt2bb; hsp70l:creb1)* transgenic fish. All the fish were subjected to heat shock for 1 h per day from 4 to 6 dpa, and the hearts were harvested at 7 dpa. We observed that the expression of *jnk1, creb1* and *c-jun* was inhibited by 73, 87, and 77% in *dn-wnt2bb* fish at 7 dpa, whereas this expression was enhanced by 51, 43, and 24%, respectively, in *wnt2bb*-overexpressing hearts (Figures 4A–C,E–G,I–K,M). As expected, the expression of *jnk1, c-jun* and *creb1 in* the *Tg(hsp70l:dn-wnt2bb; hsp70l:creb1) transgenic fish* were equivalent to that observed in the control (Figures 4A,D,E,H,I,L,P,M). We also observed that the PCNA/Mef2c proliferation index value was normal for the *Tg(hsp70l:dn-wnt2bb; hsp70l:creb1)* hearts at 7 dpa (Figures 4N–P). Furthermore, the phenotype induced by *dn-wnt2bb* could be rescued in *hsp70l*:*creb1*-overexpressing fish. These results indicate that *wnt2bb* influences CM proliferation during heart regeneration through the *jnk1/c-jun/creb1* non-canonical Wnt pathway.

### *Wnt2bb/jnk1/c-jun/creb1* Signaling Regulates Zebrafish Heart Regeneration Through *nkx2.5*

**FIGURE 5:**
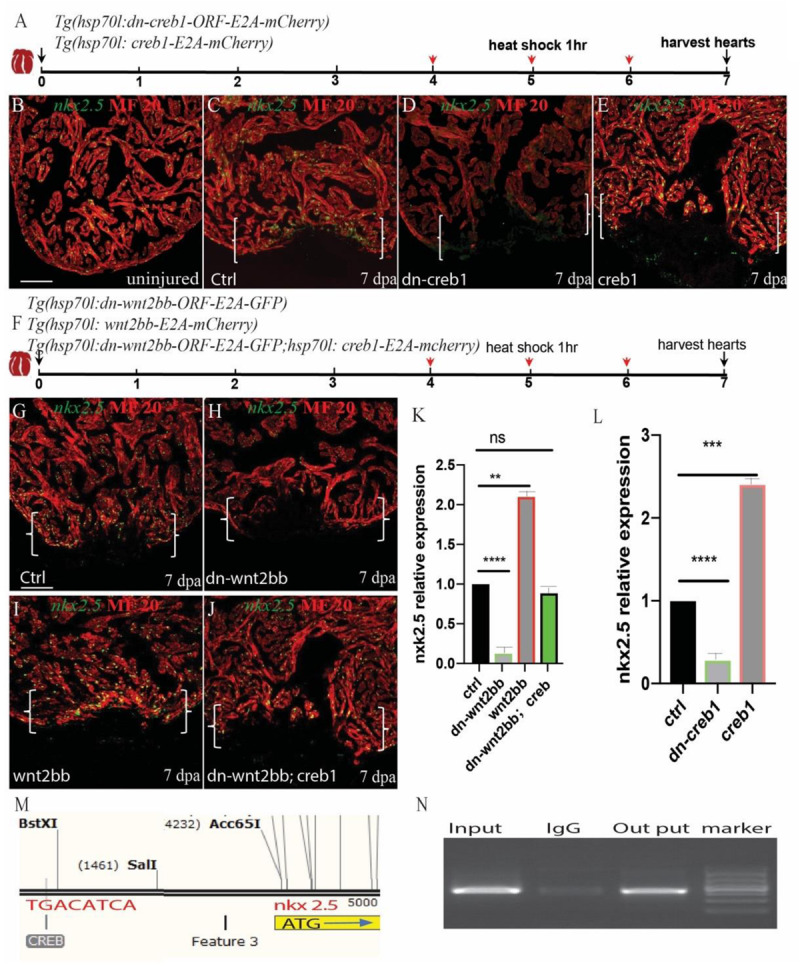
*Wnt2bb/c-jun/creb* pathway mediated zebrafish heart regeneration through *nkx2.5*. **(A,F)** Schematic of the experimental plan after apex amputation. **(B)** In uninjured hearts, *nkx2.5* was detectable in cardiomyocyte nuclei throughout the myocardium. **(C)** Following ventricular resection, *nkx2.5* was induced at the apical cell edge of wounded myocardia at 7 dpa. **(D)** *Nkx2.5* was inhibited in dn-creb hearts following ventricular resection. **(E)** *Nkx2.5* was enhanced in *creb*-ORF-overexpressing hearts following ventricular resection at the apical cell edge of wounded CMs. **(G)** Confocal image analyses displaying *nkx2.5* at wounded myocardial cell edges in control hearts **(I)**, elevated *nkx2.5* in injured *Tg(hsp70l:wnt2bb)* hearts **(H)**, diminished *nkx2.5* in *Tg(hsp70l:dn-wnt2bb)* hearts **(J)** and rescued in *Tg(hsp70l:dn-wnt2bb;hsp70l:creb1)* hearts. **(M,N)** Schematic of the CREB binding site CRE (TGACATCA) at the upstream 3.7 kb of *nkx2.5* ATG **(I)** and CHIP experiment show that *creb* can bind the CRE site of *Nkx2.5*. **(K,L)** Bar charts showing the quantification of relative *nkx2.5* expression in heat-shocked, injured wild-type (ctrl), *Tg(hsp70l:dn-wnt2bb), Tg(hsp70l:wnt2bb)*, *Tg(hsp70l:creb1)*, *Tg(hsp70l:dn-wnt2bb)*, and *Tg(hsp70l:dn-wnt2bb;hsp70l:creb)* fish hearts. The data from five hearts in each group are presented. Error bars: ± 1 SD. Significance was determined using a Student’s *t*-test: **P < 0.01, ***P < 0.001, ****P < 0.0001. Green, *nkx2.5;* Red, MF20; Brackets, amputation area; Scale bar, 100 μm. Fluorescent intensities were measured at the injury border zone using ImageJ.

CREB is a cellular transcription factor that binds to specific DNA sequences called cAMP response elements (CRE) to increase or decrease the transcription of target genes. We observed that creb1 expression was increased in the proliferating CMs. Previous research has shown that *nkx2.5* contains two CREB consensus binding sites that are essential for cardiac-specific expression in Drosophila (Venkatesh et al., 2000). Thus, we reasoned that *creb1* may bind to the promoters of some cardiac precursor cell markers, such as gata4 or *nkx2.5*, and enhance their expression. Interestingly, we observed that *nkx2.5* and *creb1* exhibited similar expression patterns in uninjured and injured hearts. The bioinformatics analysis results identified three CRE elements (TGACATCA) in the enhancer region of *nkx2.5*. We performed a CHIP experiment using an anti-creb1 antibody, and the results showed that *creb1* protein showed significant binding to the CRE-harboring region (Figures 5M,N). To confirm further that *creb1* could regulate *nkx2.5* expression, the *Tg(hsp70l:dn-creb1)*, *Tg(hsp70l:creb1)* and *Tg(hsp70l:wnt2bb; hsp70l:creb1)* transgenic fish were subjected to heat shock for 1 h every day at 37°C from 4 to 6 dpa, and the hearts were harvested at 7 dpa (Figures 5A,F). We observed that the expression of *nkx2.5* was enhanced 1.4-fold in the creb1-overexpressing hearts at the injury site (Figures 5B,C,E,L), whereas in the creb1 dominant negative mutant hearts, *nkx2.5* expression was inhibited at both the injury site and in the remote zones of the injured heart (Figures 5C,D,L). These results proved that *nkx2.5* expression is controlled by *creb1* in proliferating cardiomyocytes during zebrafish heart regeneration. Consistent with previous results, *nkx2.5* expression was reduced 87.2% in the hearts of *dn-wnt2bb* heat-shocked fish but elevated 1.1-fold when *wnt2bb* was overexpressed. Furthermore, the reduction in *nkx2.5* expression caused by *dn-wnt2bb* could be rescued by *creb1* overexpression in heat-shocked fish (Figures 5F–K). These results indicate that *wnt2bb*-mediated CM proliferation occurs through *jnk1/c-jun/creb1* non-canonical Wnt signaling.

### Non-canonical and Canonical Wnt Signaling Antagonize Each Other to Regulate Cardiomyocyte Proliferation During Zebrafish Heart Regeneration

**FIGURE 6:**
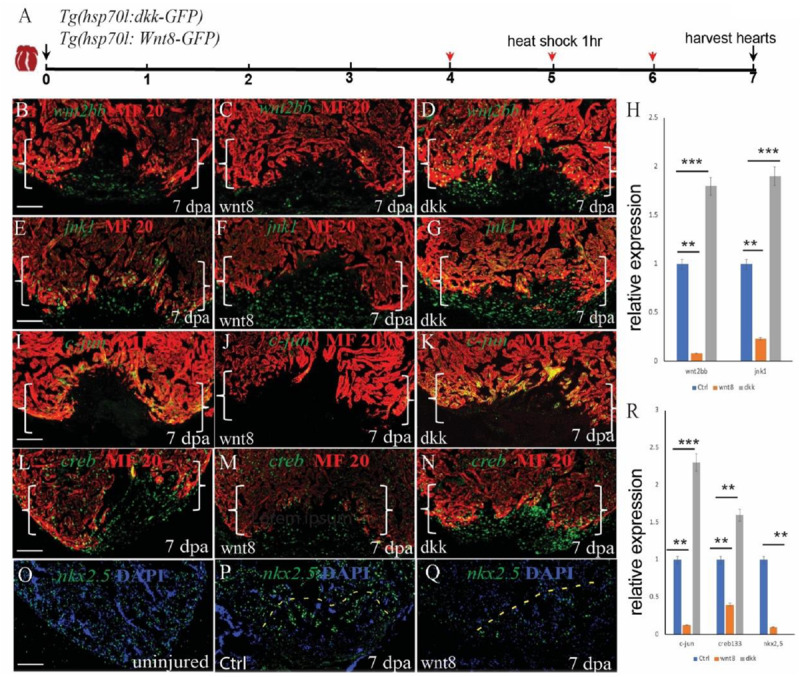
Non-canonical and canonical Wnt signaling antagonize each other to regulate cardiomyocyte proliferation during zebrafish heart regeneration. **(A)** Schematic of the experimental plan after apex amputation. **(B–D)** Immunostaining of *wnt2bb* (green) in the hearts of control, *Tg(hsp70l:wnt8)* and *Tg(hsp70l:dkk)* fish at 7 dpa. **(E–G)** Immunostaining of *jnk1* (green) in the hearts of control, Tg(*hsp70l:wnt8*) and *Tg(hsp70l:dkk)* at fish 7 dpa. **(I–K)** Confocal microscopy images of hearts from control, Tg(*hsp70l:wnt8*), and Tg(*hsp70l:dkk*) fish at 7 dpa. Sections were stained for MF20 (red) and *c-jun* (green). **(O–Q)** Confocal microscopy images of hearts from uninjured, control and *Tg(hsp70l:wnt8)* fish at 7 dpa. Sections were stained for *nkx2.5* (green). **(I–N)** Confocal microscopy images of hearts from control, *Tg(hsp70l:wnt8)*, and *Tg(hsp70l:dkk)* fish at 7 dpa. Sections were stained for MF20 (red) and *creb1* (green). **(H,R)** Bar graph showing the relative gene expression at 7 dpa in the control, *Tg(hsp70l:wnt8)* and *Tg(hsp70l:dkk)* fish hearts. Scale bars: 50 mm. Fluorescence intensities were measured at the injury border zone using ImageJ. The data from 5 to 7 hearts for each group are presented. Error bars: ± 1 SD. Significance was determined using a Student’s *t*-test: **P < 0.01, ***P < 0.001. Brackets indicate the amputation planes. Scale bar, 100 μM; Ctrl, control.

In heart development, canonical and non-canonical Wnt signaling are in balance. When canonical Wnt is signaling elevated, non-canonical signaling Wnt is low, and vice versa. To assess the effect of non-canonical wnt signaling on heart regeneration, we used the transgenic fish lines *Tg(hsp70l:dkk)* and *Tg(hsp70l:wnt8)* and induced non-canonical Wnt signaling. Consistent with the hypothesis, inhibition of canonical Wnt signaling by dkk increased the expression of *jnk1, c-jun*, and *creb1*, while increasing canonical Wnt signaling decreased the expression of *jnk1, c-jun, creb1* and *creb1* and the downstream target *nkx2.5* (Figures 6A,E–R). Interestingly, *wnt2bb* also showed crosstalk between *dkk* and *wnt8*. The expression of *wnt2bb* was impaired in *wnt8* heat-shock injured hearts and enhanced in *dkk* heat-shocked hearts (Figures 6A,B–D,H). Taken together, these results provide evidence that *nkx2.5* expression is under the control of *wnt2bb*, a Wnt ligand that induces the *jnk1/c-jun/creb1* pathway during zebrafish heart regeneration.

## Discussion

Previous studies have indicated that inhibition of canonical Wnt signaling using IWR1 promotes zebrafish CM proliferation (Zhao et al., 2019). In the present study, we showed that *wnt2bb* induced *jnk1/c-jun/creb1* non-canonical Wnt signaling in cardiomyocytes near the injury site. This result was is consistent with previous results in which inhibition of canonical Wnt signaling in *hsp70l:dkk* transgenic fish increased *jnk1/creb1* pathway signaling, and increasing the *wnt2bb*-induced non-canonical pathway enhanced CM proliferation in heart repair. In contrast, Zhao et al. (2019) activated Wnt signaling using the inhibitor BIO, which resulted in complete heart regeneration. In the current study, activating the canonical Wnt pathway in *hsp70l:wnt8* fish impaired *wnt2bb*-induced non-canonical wnt signaling. This result may explain why inhibition of canonical Wnt signaling is required for zebrafish heart regeneration.

The Wnt pathway is complex, and in invertebrates, there are 19 different Wnt proteins that are spatially and temporally regulated during development (MacDonald et al., 2009). Moreover, a specific ligand may regulate canonical or non-canonical WNT signals at different times or in different tissues. *Wnt2bb* was previously shown to function in the *Wnt2b*-mediated *Wnt/beta-catenin* pathway in small cell cancer and in other tissues (Cho and Cepko, 2006; Goss et al., 2009). A previous study showed that *wnt2* accelerates cardiac myocyte differentiation from ES-cell derived mesodermal cells (Onizuka et al., 2012). In the present study, we showed that *wnt2bb* and not *wnt2* induces *jnk1/c-jun/creb1* signaling, which participates in the non-canonical Wnt pathway in the regulation of heart regeneration.

Lepilina et al. (2006) observed that *nkx2.5*, *tbx5* and *gata4* are the earliest embryonic cardiac genes to be reactivated during zebrafish heart regeneration. In addition, Kikuchi et al. observed that the new muscle is dedifferentiated from existing cardiac myocytes during zebrafish heart regeneration (Kikuchi et al., 2010). However, the mechanism by which embryonic cardiac marker genes are reactivated remains unclear. *Nkx2.5* is one of the earliest cardiac progenitor cell markers (Dyer and Kirby, 2009), and *Drosophila* tinman/nkx2.5 mutants do not have a heart tube (Bodmer, 1995). The results of our study suggest that Wnt2bb-mediated non-classical Wnt signaling reactivated *nkx2.5* during cardiac regeneration. This discovery provides a better understanding of the mechanisms involved in cellular dedifferentiation and may aid in the development of treatments for heart diseases.

Some small molecules can activate *jnk1/c-jun/creb1* signaling. For example, forskolin is a ubiquitous activator of eukaryotic adenylyl cyclase (AC) in a wide variety of cell types and is commonly used to promote the expression of *creb1 ser133* by increasing the levels of cAMP (Trevisan et al., 2006). In addition, anisomycin is an antibiotic that inhibits protein synthesis and acts as a JNK activator (Barancik et al., 1999). These compounds could be used to improve the induction efficiency of cardiac myocytes *in vitro* or to develop treatment methods for cardiac diseases *in vivo*. In summary, the results of our study provides evidence that the wnt2bb-mediated *jnk1/c-jun/creb1* non-canonical Wnt pathway regulates cardiomyocyte proliferation through the embryonic cardiac gene *nkx2.5*, which may lead to the development of therapies for ischemic heart disease.

## Data Availability Statement

All datasets generated for this study are included in the article/Supplementary Material.

## Ethics Statement

The animal study was reviewed and approved by Guangzhou Women and Children’s Medical Centre.

## Author Contributions

XP and SF performed the experimental design and the majority of experiments, analyzed the majority of data, and drafted the manuscript. JT, ZZ, MS, and YZ performed parts of the experiments and data analyses. ZZ and LX performed parts of the experiments and image analyses. MY, XF, and LX performed patient sample enrollment and manuscript editing/review. WC and WT directed all aspects of the experiments, data analyses, and manuscript editing and review.

## Conflict of Interest

The authors declare that the research was conducted in the absence of any commercial or financial relationships that could be construed as a potential conflict of interest.
