# Supplementary material for: Wnt2bb Induces Cardiomyocyte Proliferation in Zebrafish Hearts via the jnk1/c-jun/creb1 Pathway
Source: Front Cell Dev Biol. 2020 May 25;8:323. doi: 10.3389/fcell.2020.00323 (PMC7261892; doi:10.3389/fcell.2020.00323)
Supplement: Supplementary file 1 [file Image_1.pdf]

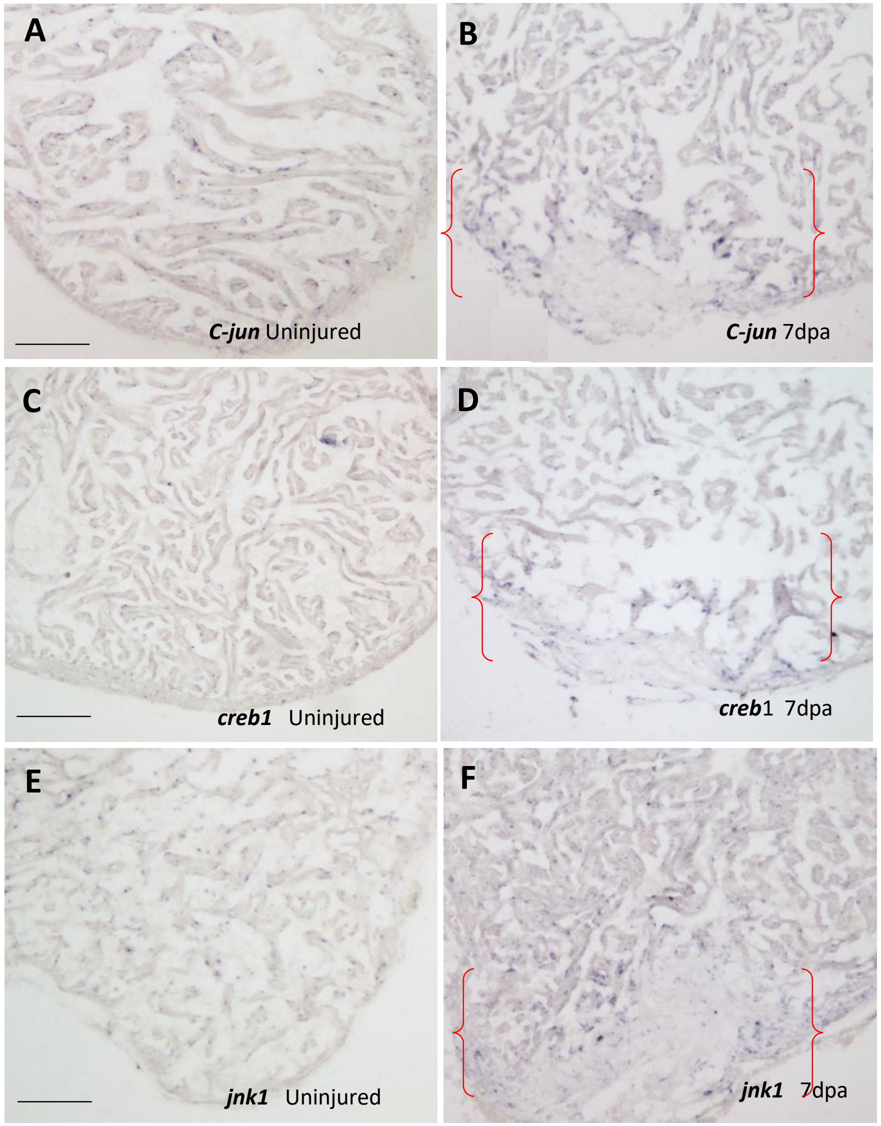

Supplementary Fig 1 . *C-jun*, *creb1* and *jnk1* expression during cardiac regeneration by ISH analyses

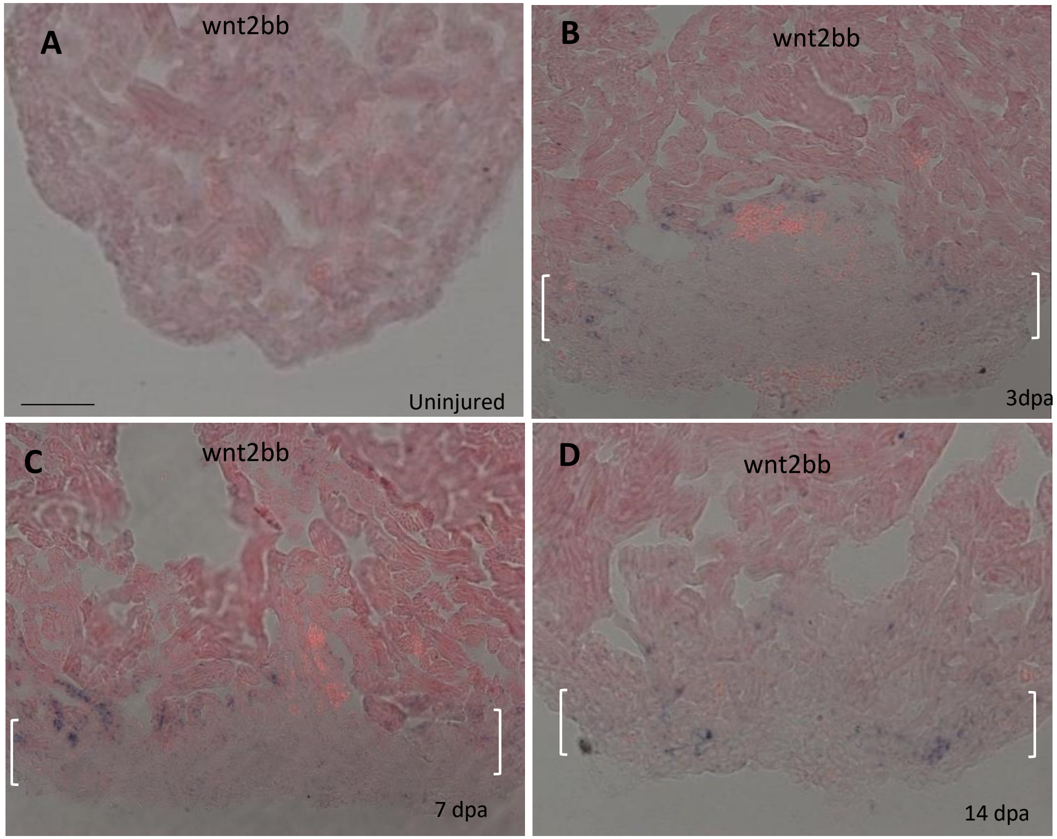

Supplementary Fig 2. *Wnt2bb* expression during cardiac regeneration by ISH analyses.

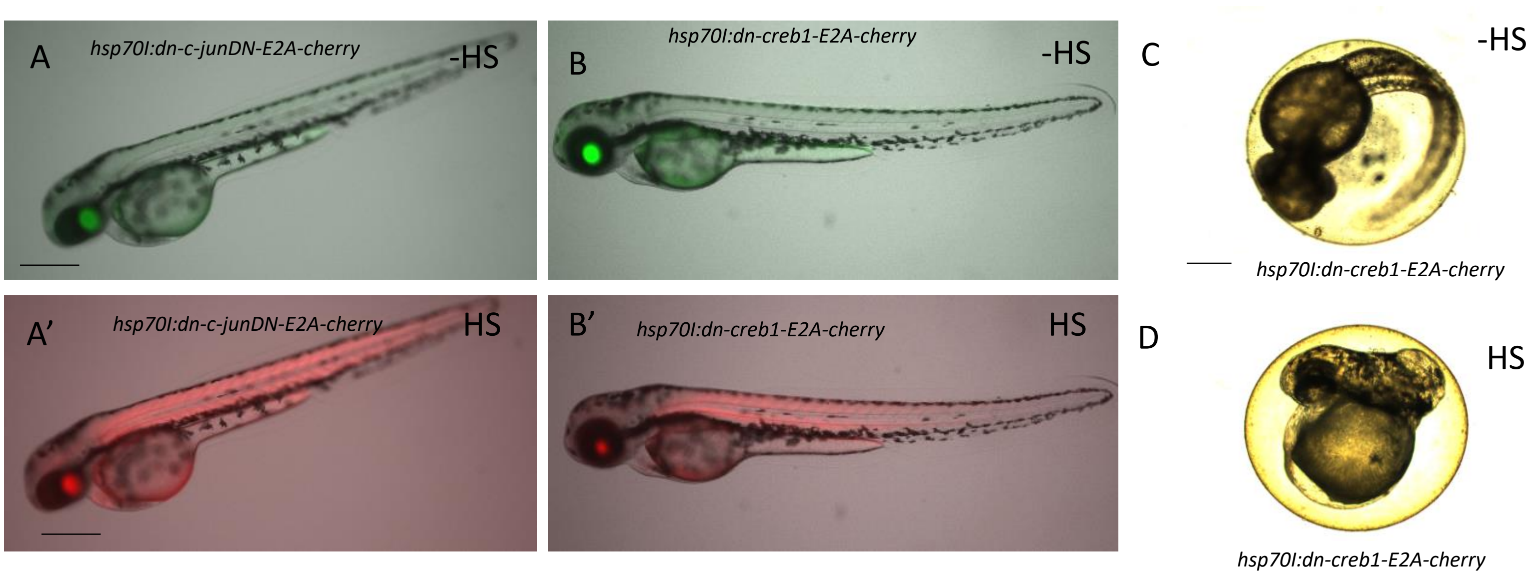

Supplementary Fig 3. Expression of *jun*, *jnk*, *creb* genes involved in noncanonical Wnt pathway analysis during cardiac regeneration.

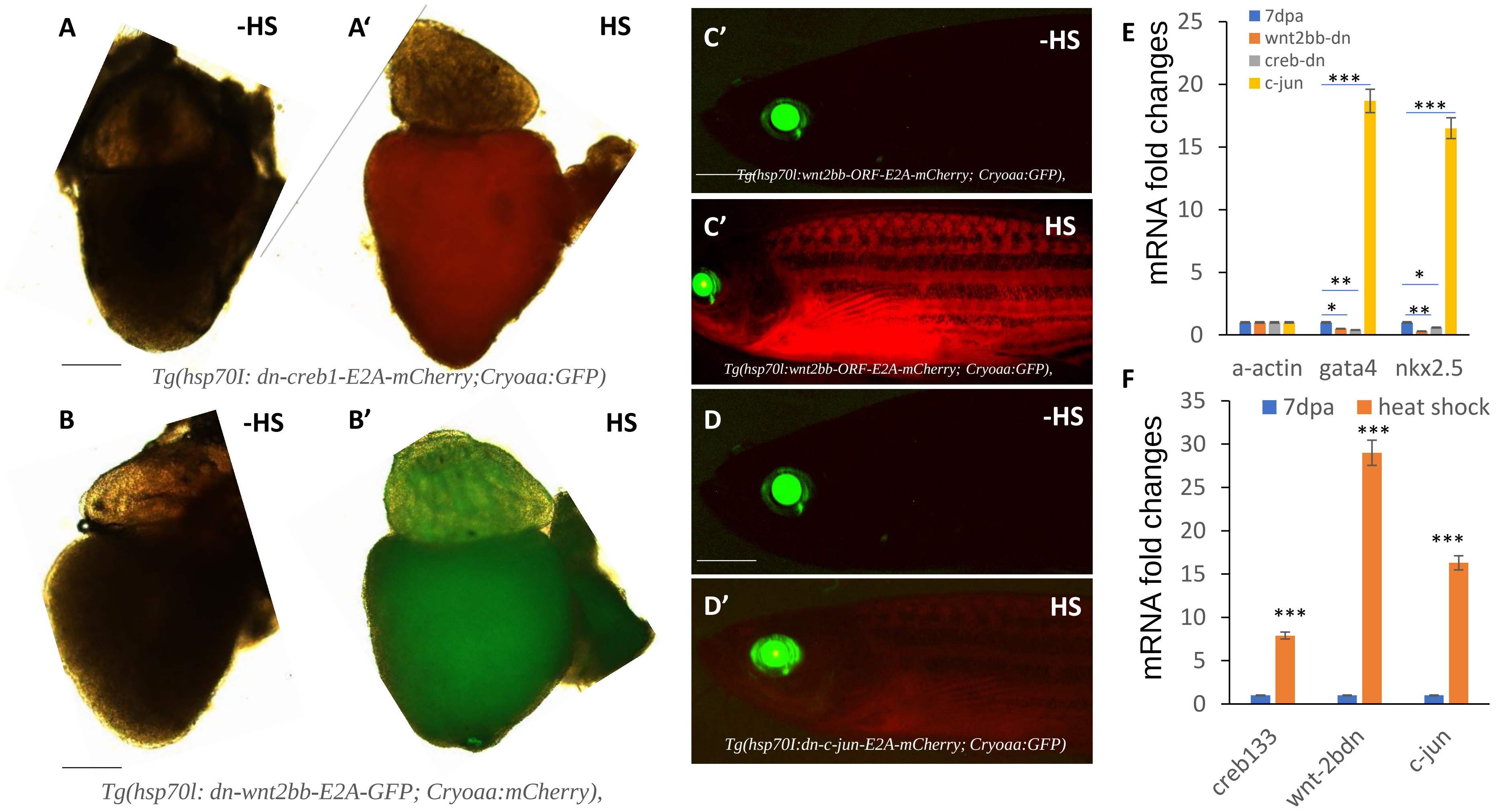

Supplementary Fig 4. Identification of transgenic fish and gene expression efficiency

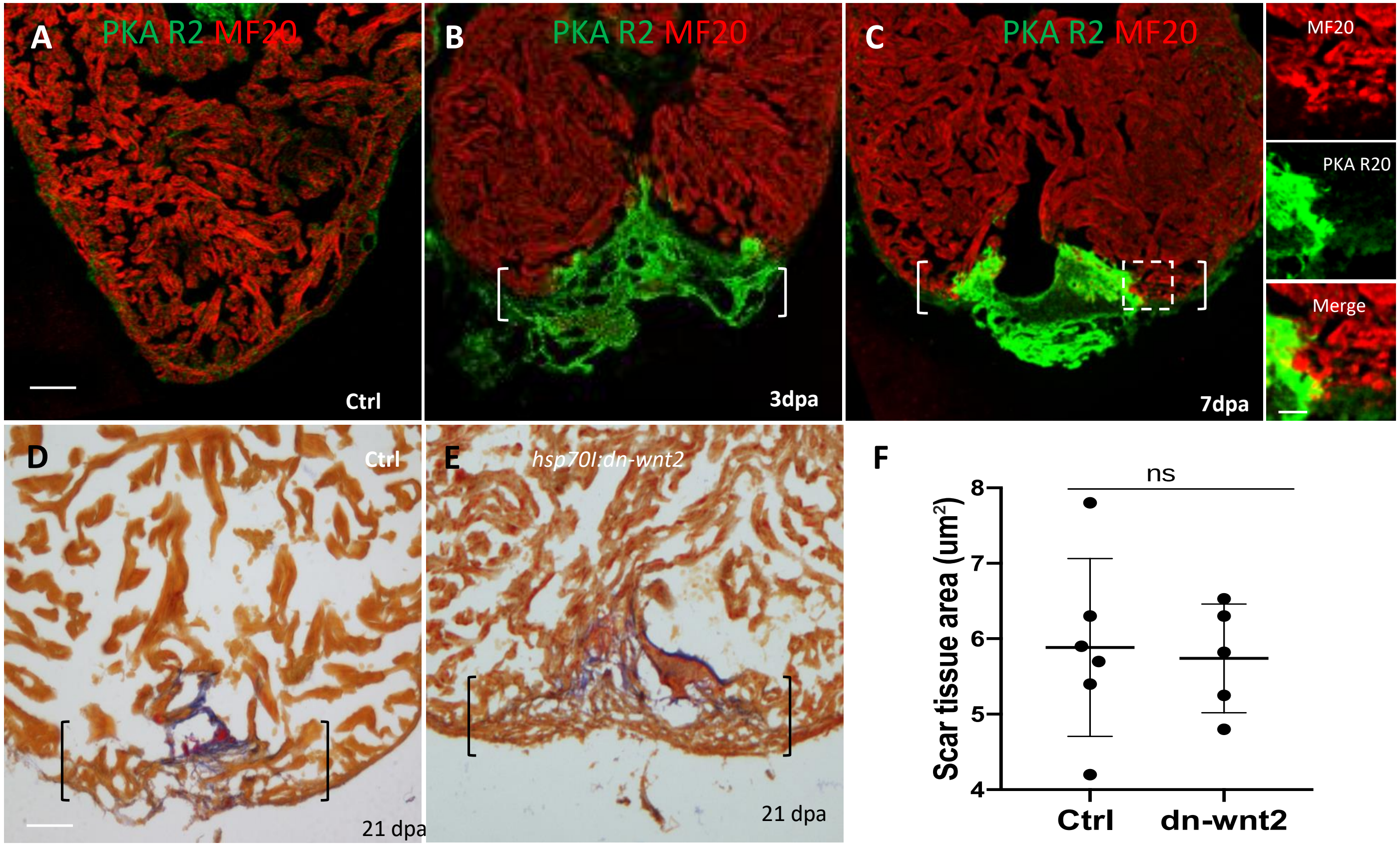

Supplementary Fig 5. ***pka*** **activated in noncardiomyocyte cells during heart regeneration.**

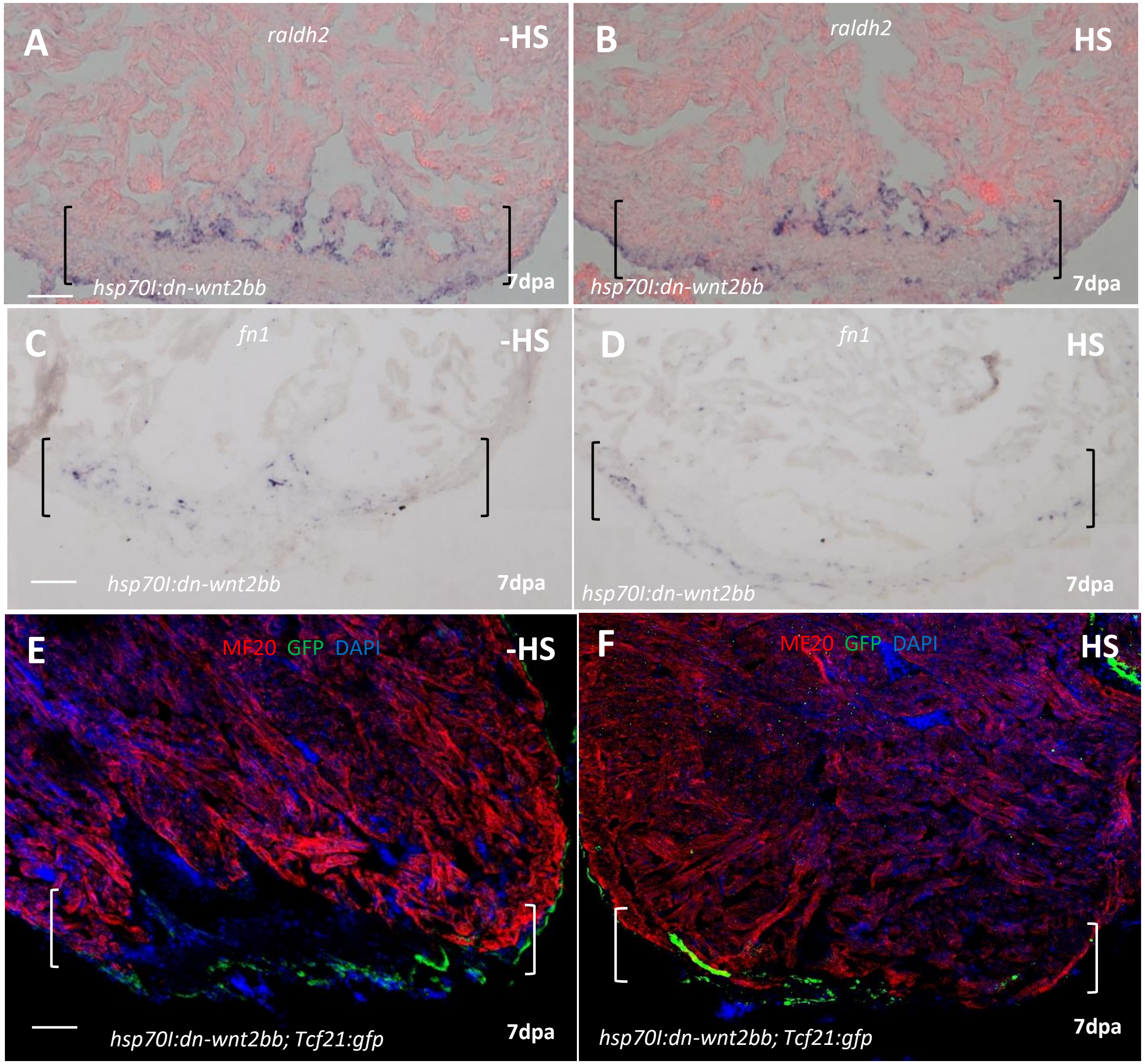

Supplement Fig 6. Wnt2bb dominant hearts have no affection on endocardium, epicardium, and fibroblasts.
